# FACTORS ASSOCIATED WITH UNMET NEED AMONG FAMILY CAREGIVERS

**DOI:** 10.1093/geroni/igz038.1417

**Published:** 2019-11-08

**Authors:** Joanne R Campione, Katarzyna Zebrak

**Affiliations:** 1 Westat, Durham, North Carolina, United States; 2 Westat, Rockville, Maryland, United States

## Abstract

This study investigates the relationship between caregiver demographics, caregiving intensity, caregiver support use, and aspects of the caregiving situation with perceived unmet need. The data source was survey response data from 1,558 informal caregivers caring for older adults (age 60+) or caring for any adult with Alzheimer’s disease or related dementia (ADRD). Participants were randomly recruited through U.S. Area Agencies on Aging client lists of any service and interviewed in December 2016. Caregivers were asked, “Are you receiving all the help you need?” Twenty-two percent (n=345) said “Definitely No” and were classified as having unmet need. We placed caregivers in tertiles based on their Zarit burden score; unmet need was 14% among low burden, 20% among medium, and 34% among high. The mean age of the care recipient (CR) was 81 years. Caregivers were 70% non-Hispanic White, 52% caring for CR with ADRD, and 43% spouse of CR. A multivariable logistic regression found two predictive factors (odds ratio p-value < 0.01) that increased the likelihood of reporting unmet need: daily intensity of caregiving and not feeling appreciated by CR. Usage of caregiver education, counseling or support group services in the past 6 months decreased the likelihood of reporting unmet need. In the high burden subgroup, Black caregivers were more likely to report unmet need than White caregivers. Understanding the relationship between caregiver demographics, self-reported burden level, service use, and the caregiver’s need for more help can assist caregiver support programs in assessing, measuring, and addressing the ongoing needs of caregivers.

